# How Does Spatial Study Design Influence Density Estimates from Spatial Capture-Recapture Models?

**DOI:** 10.1371/journal.pone.0034575

**Published:** 2012-04-23

**Authors:** Rahel Sollmann, Beth Gardner, Jerrold L. Belant

**Affiliations:** 1 Department of Environmental Resources – Fisheries and Wildlife Program, North Carolina State University, Raleigh, North Carolina, United States of America; 2 Carnivore Ecology Laboratory, Forest and Wildlife Research Center, Mississippi State University, Mississippi State, Mississippi, United States of America; University of Manitoba, Canada

## Abstract

When estimating population density from data collected on non-invasive detector arrays, recently developed spatial capture-recapture (SCR) models present an advance over non-spatial models by accounting for individual movement. While these models should be more robust to changes in trapping designs, they have not been well tested. Here we investigate how the spatial arrangement and size of the trapping array influence parameter estimates for SCR models. We analysed black bear data collected with 123 hair snares with an SCR model accounting for differences in detection and movement between sexes and across the trapping occasions. To see how the size of the trap array and trap dispersion influence parameter estimates, we repeated analysis for data from subsets of traps: 50% chosen at random, 50% in the centre of the array and 20% in the South of the array. Additionally, we simulated and analysed data under a suite of trap designs and home range sizes. In the black bear study, we found that results were similar across trap arrays, except when only 20% of the array was used. Black bear density was approximately 10 individuals per 100 km^2^. Our simulation study showed that SCR models performed well as long as the extent of the trap array was similar to or larger than the extent of individual movement during the study period, and movement was at least half the distance between traps. SCR models performed well across a range of spatial trap setups and animal movements. Contrary to non-spatial capture-recapture models, they do not require the trapping grid to cover an area several times the average home range of the studied species. This renders SCR models more appropriate for the study of wide-ranging mammals and more flexible to design studies targeting multiple species.

## Introduction

Knowledge of abundance and density of animal populations is fundamental to their conservation and consequently, methods of how to estimate these parameters have received much attention in the field of applied statistics [1]–[3]. A central issue in estimating abundance and density is that individuals cannot be observed perfectly. Capture-recapture models that use individual-level detection/non-detection data and account for imperfect individual detection [4]–[6] are one of the most popular approaches towards dealing with this problem.

Non-invasive detector arrays are becoming increasingly popular to obtain individual level detection data, particularly for cryptic and rare species. When individuals can be distinguished based on natural physical characteristics such as spot or stripe patterns, or based on artificial tags, camera traps are an ideal tool to obtain large amounts of individual-level data over large areas relatively quickly and with relatively low effort (e.g.,[7]). For species that cannot be identified individually based on visual cues, non-invasive genetic sampling in the form of hair snares or scat collection surveys can yield suitable data for capture-recapture modelling [8]–[11].

The spatial organization of traps – no matter what type – and the characteristics of the species of interest influence the data we collect. Study design has to consider both the total size of the trap array and the spacing of traps relative to individual movement. Regular (i.e. non-spatial) capture-recapture models estimate abundance, which then has to be translated into a density estimate. To do so, researchers traditionally make use of individual capture locations to estimate potential movement off the trapping grid, and apply this movement estimate as a buffer surrounding the trap array (e.g., [12], [13]). Abundance is assumed to refer to the resulting ‘effective sampled area’. Only a trapping grid that is large relative to individual movement can capture the full extent of such movements, and researchers have suggested that the grid size should be at least four times that of individual home ranges to avoid positive bias in estimates of density [14]. This recommendation originated in small mammal trapping, and it should be relatively easy to follow when dealing with species covering home ranges <1ha. However, translated to large mammal research, this can entail having to cover several thousands of square kilometres.

Trap spacing on the other hand determines the resolution of the information on individual movement [12], [13]. If trap spacing is too wide, there is little to no information on animal movement because most animals will only be captured at one trap [15]. Finally, regular capture-recapture models are based on the assumption that all individuals in the sampled area have a probability >0 of being detected, which means that trap spacing has to be narrow enough so that the array has no ‘holes’ that could contain an individual's entire home range [7]. This puts an upper limit to possible trap spacing.

All these assumptions and considerations related to regular CR models guide current recommendations for the spatial design of the trapping array in capture-recapture studies. However, more recently spatial capture-recapture (SCR) models, which explicitly describe a model of individual movement and distribution in space relative to the trap array [16], [17], have become increasingly popular to estimate population density. SCR models were initially claimed to perform well under arbitrary sampling plot sizes [17], but a recent re-evaluation suggested that small sampling plots or arrays relative to individual movement may limit the performance of SCR models [18]. So far, SCR models have not been examined thoroughly for their robustness to spatial trapping design questions.

Here we investigate how the spatial arrangement of the trapping grid influences parameter estimates for SCR models. We manipulate an empirical set of black bear (*Ursus americanus*) hair snare data to see how the size of the trap array and trap dispersion influence parameter estimates for a single species. To generalize our conclusions, we also present a simulation study examining the influence of animal movement relative to the trap array size and spacing.

## Methods

### The model

For analysis, we adopted the spatial capture-recapture model described by [17] and [19]. We assume that each individual *i* has an activity centre ***s*** *_i_*, characterized by a pair of coordinates. All ***s*** are located within the state-space *S*, an area encompassing the trapping grid, which needs to be defined as part of the model. In practice, *S* has to be chosen large enough so as to include all individuals that could have been exposed to the trapping grid. Beyond that threshold, *N* increases as *S* increases creating constant density despite a change in *S* [3], [19], contrary to non-spatial capture-recapture approaches to estimating density, where *N* is estimated independently from the sample area and thus density changes as a function of the buffer used to estimate the effective sample area.

We further assume that the observed encounter history for individual *i* at trap *j* during sample occasion *k*, *y_ijk_*, are mutually independent outcomes of a Bernoulli random variable
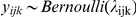
where variation in *λ_ijk_* is modeled such that



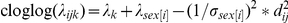

*d_ij_* is the distance between the activity centre for individual *i*, ***s_i,_*** and the location of trap *j*. *σ_sex_* (units of the trapping grid, here km) controls the shape of the decreasing function of detection as *d_ij_* increases (i.e., the probability of being detected in a trap decreases as the distance from the activity centre to the trap location increases). This declining function is an approximate half-normal distribution, and assuming a bivariate normal movement model (i.e., a circular home range with movements concentrated around the centre), we can translate *σ* to an estimate of the radius of the home range occupied during the study period [20]. We expect that movement is different between the sexes and thus allow males and females to have separate *σ*.

This form, the complementary log-log relationship, is the result of reducing a Poisson encounter model, which would be used in the case when an individual can be captured multiple times in multiple traps during one sampling occasion. Here, *λ_sex_* is the – sex-specific – baseline trap encounter rate and *λ_k_* is the occasion specific encounter rate, which is the encounter rate if an activity centre is located precisely at a trap, i.e., when *d_ij_*  = 0.

To estimate *N*, the population size in *S*, we used a Bayesian analysis by data augmentation of the model [21]. In data augmentation, we let *M* be a number that is larger than the largest possible population size (i.e., the number of activity centres, *N*) in *S*, and *n* be the number of detected individuals. We assume a prior distribution for *N* that is uniform over the interval (0, *M*) and augment the observed data set with *M* – *n* individuals who were never detected and thus have encounter histories that are all zero. This reformulation of the model based on data augmentation is a zero-inflated binomial mixture and the number of activity centres *N* in *S* is then estimated as a fraction of *M*. The motivation for data augmentation is to fix the size of the data set at *M*, instead of using the unknown *N*, which is computationally advantageous when implementing the model in a Bayesian framework using Markov chain Monte Carlo (MCMC) sampling [21]. *M* is adequately large when estimates of *N* are not limited by, *M*.

### Data: Black bears from Pictured Rocks National Lakeshore

**Figure 1:**
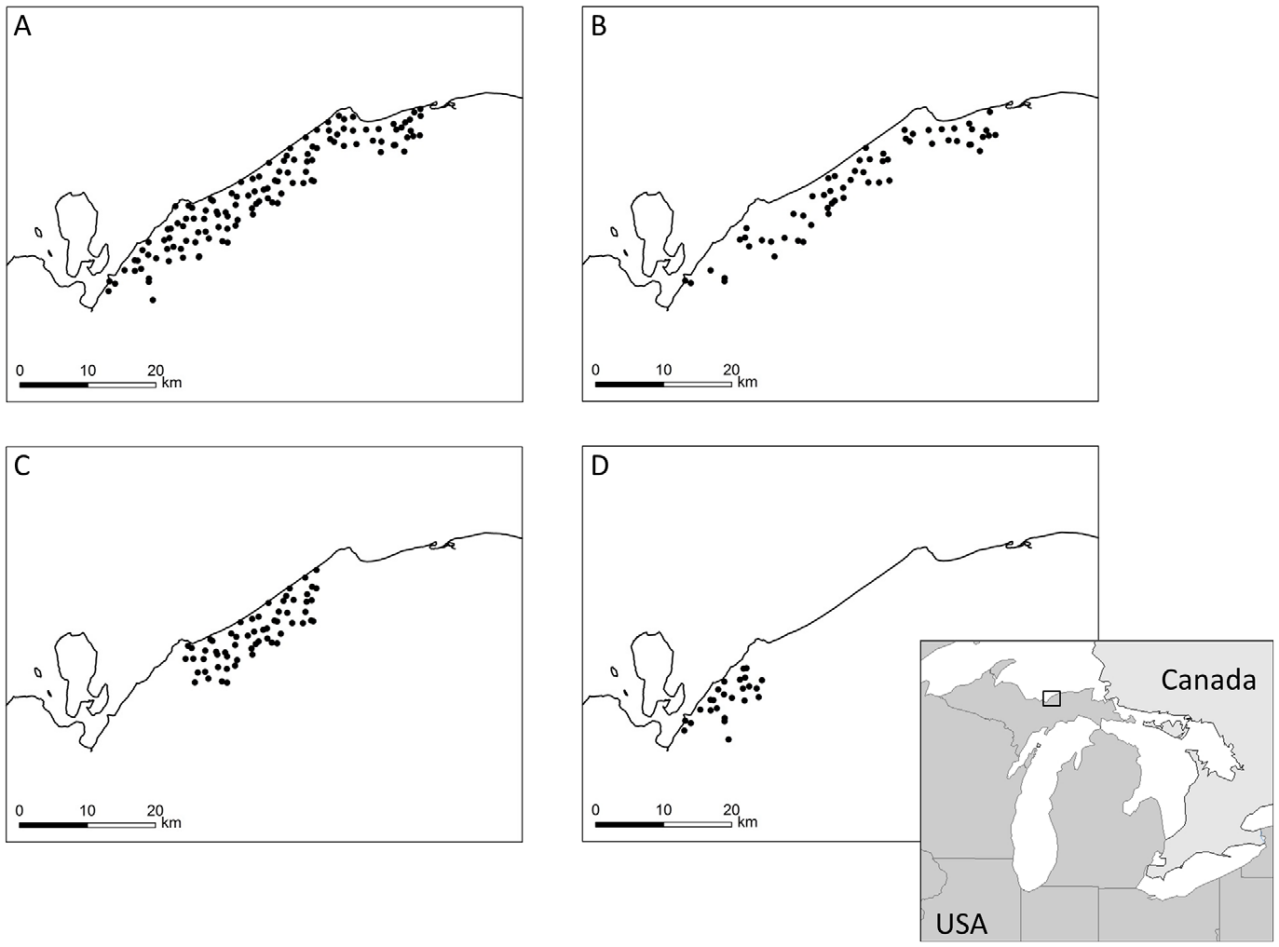
Location of the study area. Location of the study area in Pictured Rocks National Lakeshore, Michigan (rectangle in inset map), and state-space with trap array layouts for the full (A) and three reduced sets of black bear hair snare data; B) 50% of all traps chosen at random; C) 50% of traps in the centre of the grid; and D) 20% of traps in the South of the grid.

Black bears were studied in the Pictured Rocks National Lakeshore, Michigan, using 123 hair snares distributed over an area of 440 km^2^ along the shore of Lake Superior (Figure 1) in May–July 2005. Hair snares were established and checked four times at 14-day intervals following [22]. Hair samples were genetically analysed using 6 microsatellite loci to determine individuals [23]; sex was determined using size polymorphism in the amelogenin gene [24]. The resulting data were used to construct encounter histories for each individual at each trap for each occasion, thus creating an *M* by *J* by *K* array. To carry out the data augmentation, 450 – *n* all-zero encounter histories were added to the encounter array. Setting *M* = 450 ensured that estimates of *N* were not limited by *M*. As described above, we formulated the model such that the baseline trap encounter *λ* rate was sex-specific and for both sexes changed in parallel over time (i.e., while *λ* changed over time, the difference in *λ* between males and females stayed constant). This was motivated by the lower average number of detections for male bears and the overall decreasing number of detections over time in the raw data. We further allowed the movement parameter *σ* to be sex-specific, as male black bears are known to move over larger areas than females (e.g., [19], [25]). We defined the state-space *S* as the outermost coordinates of the trapping array plus a 15-km buffer (preliminary analyses showed that a further increase in buffer width did not lead to changes in density, and we thus concluded that 15 km was appropriate). The resulting rectangle included parts of Lake Superior (Figure 1). To account for the fact that black bears do not live in the lake we constrained individual activity centres to the terrestrial portion of *S,* thus resulting in a state space of size 2525 km^2^. To estimate density, we divided the estimate of *N* by the area of *S,* to get the number of individuals per 100 km^2^. Considering that most studies are limited by the number of available traps, we wanted to understand whether the spatial arrangement of traps influences model parameter estimates. To address the impact of a smaller trap array on the parameter estimates, we first calculated the geographic centre of the trapping array and retained only those 50% of the traps closest to the grid centre. To examine the impacts of a trap array that is small relative to individual movement, we then removed 80% of all traps, retaining only the southern 20% of the trap array. Finally, to represent a setup where traps are spaced wider to achieve larger area coverage, which should result in a larger number of individuals exposed to trapping, we randomly removed 50% of the traps and performed model analysis only with data from the remaining traps (Figure 1). For all scenarios we retained the *S* we defined for the full data set, so that all estimates of *N* refer to the same area. If SCR models are not sensitive to trap spacing and the size of the sampled area, we expect estimates of movement, detection and density to remain similar across scenarios. Slight fluctuations in parameter estimates can be expected since the scenarios involve different subsets of the black bear population. However, we did not expect true bear density to vary among the different scenarios since habitat conditions were relatively uniform across the trap array. We used parameter estimates from the full data set as reference for comparisons among scenarios.

### Model implementation

We implemented the analysis using the program WinBUGS [26] accessed through the software R [27] with the package R2WinBUGS [28]. WinBUGS uses Gibbs sampling, a Markov chain Monte Carlo (MCMC) method simulating samples from the joint posterior distribution of the unknown quantities in a statistical model [29]. MCMC chains are started at arbitrary parameter values and since successive iterations depend on the outcome of the previous iteration, the start value will be reflected in a number of initial iterations that should be discarded (the burn-in). All models were run for 10000 iterations, with a burn in of 5000, with three chains and a thinning rate of 3. We checked for chain convergence using the Gelman-Rubin statistic [30], R-hat, which compares between and within chain variation. R-hat values below 1.1 indicate convergence [31]. Values for all estimated parameters for the empirical and the simulated data were below 1.1.

## Results

With exception of the 20% area scenario, results were similar across the different trap arrays. In general, relative to the full dataset, the reduced datasets did not perform as well. Particularly, the movement parameter *σ* was underestimated for males, and so was the difference in detection between males and females. Density estimates were included in the 95% BCI of the full dataset. Only for the 20% area scenario, difference in detection between genders was overestimated and differences in parameter estimates translated into a significantly lower density estimate.

### Full data set

The full data set consisted of 393 captures of 38 females and 45 males. On average, females were captured more often than males (5.50 and 4.09 times, respectively). Female baseline trap encounter rate *λ* decreased over time from 0.47 ±0.06 SE in the first occasion to 0.16 (±0.03 SE) in the last occasion; males had lower *λ_k_*, reflected by a negative coefficient for being a male (*β* (male)) in the log-linear predictor of *λ_k_* (Table 1). With 2.94 km (± 0.14 SE), *σ* for females was less than half of *σ* for males (7.45 km ± 0.50 SE). The sex ratio was slightly skewed towards males, but with a 95 % Bayesian Credible Interval (95BCI) including 0.5 it did not diverge significantly from a 1∶1 ratio. Density was estimated at 10.56 individuals 100 km^−2^ ± 1.08 SE.

### Grid area – 50% of all traps

After removing the outermost 50 % of all traps, the data set consisted of 208 captures of 21 females and 25 males. On average, females were captured 4.86 times, and males 4.24 times.

Overall, the baseline detection was smaller when compared with the full data set, and there was less difference between males and females (Table 1). The time trend in *λ_k_* remained similar, although there was a slight increase from occasion 1 to 2 before *λ_k_* decreased (Table 1). With 3.32 km (± 0.28 SE) *σ* for females remained similar. *σ* for males was significantly lower than in the full data set (5.35 km ± 0.51 SE). Sex ratio did not diverge from 1∶1. The density estimate was slightly higher than in the full data set, with 12.65 individuals 100 km^−2^ (± 1.84 SE).

### Grid area – 20% of all traps

Retaining only 20 % of all traps, the data set consisted of 103 detections of 12 males and 13 females. Females were captured on average 5.31 times and males 2.83 times.

*λ_k_* almost doubled for females compared to the full data set and the difference between males and females increased. Also, the decrease in *λ_k_* over time was weaker (Table 1). With 9.88 km (± 3.57 SE), *σ* for males was significantly larger than in the full data set and had large confidence limits (Table 2), while *σ* remained similar for females (2.69 km ± 0.39 SE). Density was 6.75 individuals 100 km^−2^ (± 1.61 SE) – significantly lower than in the full data set. The sex ratio shifted more towards females, but was not significantly different from 1∶1.

### Trap spacing – 50% of all traps

After randomly removing 50% of the traps, the data set consisted of 201 captures of 31 females and 35 males. The average number of captures remained higher for females than males (3.42 and 2.71, respectively). We found effectively no differences in parameter estimates between this and the full data set (Table 1 and 2).

**Table 1:** Estimates of baseline trap encounter rates *λ_k_* (SE) for black bears sampled with hair snares.

	Occasion 1	Occasion 2	Occasion 3	Occasion 4	*β*(male)
Full data set	0.47 (0.06)	0.41 (0.06)	0.22 (0.04)	0.16 (0.03)	−1.46 (0.17)
Grid area –50 %*	0.37 (0.07)	0.39 (0.08)	0.18 (0.04)	0.07 (0.02)	−0.61 (0.25)
Grid area –20 %**	0.87 (0.28)	0.98 (0.29)	0.55 (0.18)	0.53 (0.19)	−2.23 (0.39)
Trap spacing***	0.44 (0.10)	0.42 (0.09)	0.22 (0.06)	0.15 (0.04)	−1.26 (0.26)

Estimates of baseline trap encounter rates *λ_k_* (SE) for black bears sampled with hair snares over four 14-day occasions in Pictured Rocks National Lakeshore, Michigan, estimated with spatial capture-recapture models for the full data set and three reduced data sets. Values correspond to female *λ_k_*, *β*(male) is the effect of being a male on *λ_k_* on the log-scale.

* Reduced trap array area – 50% innermost traps used.

** Reduced trap array area – 20% southernmost traps used.

*** Increased trap spacing by random removal of 50% of the traps.

**Table 2:** Posterior summaries of SCR model parameters for black bears.

Data set	Parameter	Mean (SE)	Mode	2.5%	97.5%
Full data set	*D* [ind./100 km^2^]	10.56 (1.08)	10.45	8.59	12.79
	*σ* (males) [km]	7.45 (0.50)	7.32	6.58	8.50
	*σ* (females) [km]	2.94 (0.14)	2.94	2.67	3.23
Grid area – 50%*	*D* [ind./100 km^2^]	12.65 (1.84)	12.21	9.31	16.71
	*σ* (males) [km]	5.35 (0.51)	5.25	4.47	6.47
	*σ* (females) [km]	3.32 (0.28)	3.26	2.84	3.91
Grid area – 20%**	*D* [ind./100 km^2^]	6.75 (1.61)	5.95	4.00	10.22
	*σ* (males) [km]	9.88 (3.57)	7.57	5.12	18.45
	*σ* (females) [km]	2.69 (0.39)	2.66	2.12	3.40
Trap spacing ***	*D* [ind./100 km^2^]	10.25 (1.18)	10.29	8.08	12.63
	*σ* (males) [km]	6.26 (0.62)	6.07	5.19	7.58
	*σ* (females) [km]	3.28 (0.26)	3.26	2.82	3.82

Posterior summaries of SCR model parameters for black bears sampled with hair snares over four 14-day occasions in Pictured Rocks National Lakeshore, Michigan, for the full data set and three reduced data sets. *D* = density, *σ* = movement parameter.

* Reduced trap array area – 50% innermost traps used.

** Reduced trap array area – 20% southernmost traps used.

*** Increased trap spacing by random removal of 50% of the traps.

### Simulation study

#### Design

To further investigate how trap spacing and array size relative to animal movement influences SCR parameter estimates, we simulated detection histories on an 8×8 trap array with regular trap spacing of 2 units. Since in reality traps are rarely set in a perfect grid, we randomly placed traps within the grid cell and the locations were newly drawn for each simulated dataset. We used the same model as described in the model section but without the time and sex-specific covariates. The complementary log-log form of the model is thus
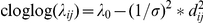
We chose four values for *σ* so that we had a scenario where the trap array was smaller than a single individual's home range (*σ* = 5 units), a scenario where spaces between traps were large enough to contain entire home ranges (*σ* = 0.5 units), and two intermediate scenarios where sigma was smaller (*σ* = 1 unit) and larger (*σ* = 2.5 units) than the trap spacing, respectively. We defined the state space as the trapping array plus a buffer of 3 times *σ* around it. Since density and home range size are often negatively correlated (e.g., [32], [33]), we chose an *N* of 100 for all four scenarios of *σ*. In combination with the varying state space this led to a decrease in density with increasing *σ*. For all scenarios we used a baseline trap encounter rate *λ_0_* of 0.5 and simulated trap encounters over 4 occasions to produce 100 data sets. Each model was run for 8000 iterations, with three chains, a burn-in of 5000 and a thinning rate of 2. We checked for chain convergence in each simulation using the Gelman-Rubin statistic [30]. For each data set we calculated the mean, standard error (SE) and mode of the posterior, as well as the relative bias (RB) and the relative root mean squared error (rrmse) of the mean, and determined whether the 95BCI included the true parameter value (BCI coverage). We report results as the average over all simulations.

#### Results

All model parameters were identifiable and estimated with relatively low bias (<10%) and high to moderate precision (rrmse<25%) for all scenarios of *σ*, except *σ* = 0.5 units (Table 3). Data for the latter case mostly differed from the other scenarios in that fewer animals were captured and very few of the captured animals were recorded at more than 1 trap (see Appendix S1 for summary statistics of the simulated data sets). For *σ* = 0.5, abundance (*N*) was not identifiable in 88 % of the simulations, and when identifiable, was underestimated by approximately 50%. Thus, we omitted parameter estimates for *σ* = 0.5 scenarios from the results but note that this was an important scenario in that a trap spacing that is considerably too large may be problematic.

Estimates of *N* and *σ* were least biased under the *σ* = 2.5 scenario, while estimates of *λ_0_* were least biased under the *σ* = 5 scenario. Precision for estimates of *N* was highest under the *σ* = 2.5 scenario, while *λ_0_* and *σ* were more precise at *σ* = 5. All estimates had the highest relative bias and the lowest precision under the *σ* = 1 scenario.

**Table 3:** Summary statistics for spatial capture-recapture parameters across 100 simulations for four simulation scenarios.

Scenario	Parameter	Mean	rrmse	Mode	2.5%	97.5%	RB	BCI
***σ*** ** = 1**	*N*	108.50	0.17	104.10	78.98	143.41	0.09	96
	*λ_0_*	0.52	0.25	0.48	0.30	0.75	0.04	94
	*σ*	1.008	0.09	0.99	0.86	1.20	0.01	94
***σ*** ** = 2.5**	*N*	100.27	0.11	98.46	82.09	121.88	<0.01	97
	*λ_0_*	0.51	0.12	0.50	0.41	0.62	0.01	92
	*σ*	2.50	0.05	2.49	2.27	2.69	<0.01	92
***σ*** ** = 5**	*N*	102.86	0.14	100.76	77.40	130.02	0.03	88
	*λ_0_*	0.51	0.08	0.50	0.44	0.58	0.01	93
	*σ*	5.02	0.04	5.00	4.69	5.43	0.01	97

Mean, relative root mean squared error (rrmse) of the mean, mode, 2.5% and 97.5% quantiles, relative bias of mean (RB) and 95BCI coverage (BCI) for spatial capture-recapture parameters across 100 simulations for four simulation scenarios, define by the input value of movement parameter *σ*. *N* = number of individuals in the state space; *λ_0_* = baseline trap encounter rate.

## Discussion

When designing a capture-recapture study for a single species, trap spacing and the size of the array can (and should) be tailored to the spatial behaviour of that species to ensure adequate data collection. However, some trapping devices like camera traps may collect data on more than one species and researchers may want to analyse these data, too. Independent of the trapping device used, study design will in most cases face a limit in terms of the number of traps available or logistically manageable. In regular CR models density estimates can be heavily influenced by trap spacing [15], [34] and the size of the trap array relative to animal movement [14]. Our evaluation of spatial capture-recapture (SCR) models, especially within the simulation study, showed that this relatively new class of models performs well across a wide range of spatial setups and animal movements.

### Array size relative to animal movement

For the black bear study, removal of 50% of the traps, either random or area-based, did not greatly influence model results. The observed smaller differences in parameter estimates (*σ* for males, and *λ_k_*) may be due to individual differences in detection and movement that manifest themselves when only a smaller portion of the overall population is sampled. For both 50% scenarios, the number of individuals sampled was lower than in the full data set, but the effect was stronger when reducing the sampled area. Also, by reducing the number of traps we effectively reduced the size of the overall data set estimates were based on (both in terms of individuals captured and recaptures). This was reflected in overall higher SE and wider confidence intervals. In spite of these differences, density estimates for black bears – the main objective of applying SCR models – remained largely constant.

Specifically, our results showed that when using SCR models, there is little need to sample areas several times an individual home range in order to obtain realistic movement estimates, as is required for non-spatial models [14]. Reducing the area of the trap array by 50% created a grid polygon of 144 km^2^, which was smaller than an estimated male black bear home range and only 50% larger than a female black bear home range – approximately 260 km^2^ and 100 km^2^ during the study, respectively, when converting estimates of *σ* to home range size. Black bear home range size varies substantially throughout North America. The largest ranges of 227 km^2^ and 606 km^2^ for females and males, respectively, have been reported from the northern Lower Peninsula in Michigan [35], while the smallest estimate comes from Washington (3.4 km^2^ for a female monitored over 60 days; [36]). Thus our model based estimates of home range size for the study duration fall within the general size range observed for the species.

It is worthy to note that in the present case the trap array resembled a narrow rectangle or ellipse, with a much larger extension in one dimension than the other (53 km as compared to 9 km at the widest point, respectively). As a consequence, even in the reduced area scenarios, the largest extent of the trap array was still larger than individual bear movement, which probably contributed to movement parameters being estimable. Although we did not explore the effect of the shape of the trap array in the present study, this may be an interesting focus for future work.

Results from our simulation study corroborate the observation that SCR models perform well even when using a trapping array smaller than an average home range: at *σ* = 5 units, the home range of an individual was approximately 235 units^2^, while our trapping grid only covered 196 units^2^. Still, the model performed well (Table 3, *σ* = 5).

The ability of SCR models to estimate movement even for relatively small trapping grids lies within the model itself: *σ* is estimated as a specified function of the ancillary spatial information collected in the survey and the capture frequencies at those locations. This function is able to make a prediction across distances and when there are latent distances (i.e., a distance larger than the extent of the trap array) the model makes a prediction of the detection at such a distance. As long as there are enough data across at least some range of distances, the model does quite well at making predictions at unobserved distances. Non-spatial CR models do not fit a function to the distances and thus are susceptible to underestimating movement when the trap array is not large enough. However, if the trap array is so small that individuals are captured with equal probability at all observed distances, then the SCR models may overestimate movement significantly. Preliminary simulation results indicate that for *σ*≤20 units parameter estimates remained largely unbiased. More research is needed regarding model performance under such extreme cases, but this again suggests that while the extent of the trap array should resemble the extent of individual movement, it does not need to be several times larger.

An important consideration in our simulation study was that all but the *σ* = 0.5 units scenarios provided large amounts of data, including 20+ individuals being captured on the trapping grid. When dealing with real-life animals that are often territorial and may have lower trap encounter rates, a very small grid compared to an individual's home range may result in the capture of few to no individuals. In that case, the sparse data will limit the ability of the model to estimate parameters [18], which is true of most models.

This is also suggested by the black bear study. Although black bears are not strictly territorial in the traditional sense, they do space themselves across a landscape and may have core home range areas that show little overlap with other individuals (e.g., [37], [38]). As a result, removing 80% of the traps and thereby reducing the area of the trap array to 64 km^2^ – well below the average black bear home range – had a great effect on sample size (only 25 of the original 83 individuals sampled) and thus parameter estimates. Particularly, male black bear movement was overestimated and imprecise (Table 2). The combination of the low baseline trap encounter rate of males and the considerable reduction in sample size led to a low level of information on male movement: five of the 12 males were captured at one trap only. Although they moved over smaller areas, owing to their higher trap encounter rate females were, on average, captured at more traps (3.4 traps per individual compared to 2.6 for males) so that their movement estimate remained relatively accurate. Overestimated male movements and female trap encounter rates resulted in an underestimate of density of almost 40%. This effect is contrary to what we would expect to see in non-spatial CR models, where too small an area leads to underestimated movement and overestimated density [14], [15], [34].

In spite of these limitations, our results show that particularly for large mammal research SCR models have much more realistic requirements in terms of area coverage than non-spatial CR models, under which density estimates can be largely inflated with small trapping grids relative to individual movement [34]. How large the spatial survey effort needs to be does not only depend on the extent of movement of the target species, but also on the temporal effort, density and detection probability [18]–in summary, the amount of data that can be collected with any given trap array.

### Trap spacing relative to animal movement

Overall, results from the reduced area model differed more from the full data set than results from the increased trap spacing model. This suggests that if logistics permit, results may benefit from spacing traps a little wider to sample a larger area – thereby exposing more individuals to sampling. It is important to note that randomly removing 50% of the traps only slightly increased the average distance between neighbouring traps, from approximately 1 to 1.2 km. Considering the movement range of black bears, with *σ* of 3–9 km (depending on the data set and sex), this change in trap spacing is negligible. Even further removal of traps would not have increased trap spacing enough to be relevant relative to black bear movement. Our simulation study allowed us to examine the effects of large trap spacing relative to animal home range size. SCR models performed well as long as *σ* was at least ½ the average distance between traps. At this trap spacing to movement ratio, most individuals are captured at one trap only (see Appendix S1). This scenario represents a problem in non-spatial CR models when estimates of the effective sampled area are based on individual movements between traps (for example, half the mean maximum distance moved between traps by individuals captured at more than one trap – [13]), as estimates of average movement tend to increase with the number of recaptures [39], [40]. Yet, with SCR models, parameter estimates exhibited low bias and remained relatively precise (see simulation results for *σ* = 1 in Table 3). In the simulation, only when *σ* = 0.5 so that the home range size is very small relative to the trap spacing and hardly any individuals were captured at more than one trap, SCR models were unable to estimate *N*. A potential way to address this issue could be a nested trapping grid with narrower trap spacing for some subset of the traps. Further research is needed to see how effective this would be within the context of other constraints such as total number of traps available, logistics, and heterogeneity in habitat.

### Conclusion

While there are limits to the flexibility in spatial trap array design for SCR modelling, the method proved much more robust to changes in trap array size and spacing relative to animal movement than non-spatial CR models. Trapping grids with an extent of approximately a home range diameter can – in theory – adequately estimated density and home range size. The ability to give reliable estimates of individual movement and density will depend on the amount of data collected (both in terms of individuals and spatially spread-out recaptures), and for highly territorial or hard-to-trap species, larger grids may be necessary to collect sufficient data. Thus, while a trap array designed for one species will not necessarily yield suitable data for other species, the flexibility of SCR models regarding spatial study design makes finding a compromise in study design for several species easier.

With their ability to extract information about animal movement and density even from smaller trap arrays, SCR models are better suited for the study of wide ranging mammals, where placing traps throughout areas of several times the average home range is usually not feasible. However, our results should not encourage researchers to design non-invasive trap arrays based on minimum area and spacing requirements. Study design should still strive to expose as many individuals as possible to sampling and obtain adequate data on individual movement. Large amounts of data can also improve precision of parameter estimates – the density estimate for the full black bear data set has narrower confidence intervals than estimates from the reduced data sets. This is particularly important when a study is concerned with monitoring population changes. Also, only with sufficiently large data sets potentially important covariates (such as gender or time effects in the black bear example) can be included into SCR models to obtain density estimates that reflect the actual state of the studied population.

## Supporting Information

Appendix S1 **Summary statistics of 100 simulated data sets for four simulation scenarios, defined by the input value of movement parameter σ.** Individual detection histories were simulated on an 8×8 trap array with regular trap spacing of 2 units under the spatial capture-recapture model described in the Simulation Study section.
(DOCX)
