# Supplementary material for: How Does Spatial Study Design Influence Density Estimates from Spatial Capture-Recapture Models?
Source: PLoS One. 2012 Apr 23;7(4):e34575. doi: 10.1371/journal.pone.0034575 (PMC3335117; doi:10.1371/journal.pone.0034575)
Supplement: Appendix S1 — Summary statistics of 100 simulated data sets for four simulation scenarios, defined by the input value of movement parameter σ. Individual detection histories were simulated on an 8×8 trap array with regular trap spacing of 2 units under the spatial capture-recapture model described in the Simulation Study section. (DOCX) [file pone.0034575.s001.docx]

Appendix SI

Summary statistics of 100 simulated data sets for four simulation scenarios, define by the input value of movement parameter *σ*. Individual detection histories were simulated on an 8 x 8 trap array with regular trap spacing of 2 units under the spatial capture-recapture model described in the Simulation Study section.

| Scenario | No. individuals captured | No. captures | No. individuals recaptured | No. individuals captured at > 1 trap |
| --- | --- | --- | --- | --- |
| *σ* = 0.5 | 18.29 (3.836) | 25.38 (5.856) | 5.52 (2.032) | 0.72 (0.954) |
| *σ* = 1 | 37.70 (13.436) | 69.35 (26.047) | 19.48 (7.684) | 11.87 (5.434) |
| *σ* = 2.5 | 44.19 (4.667) | 231.78 (33.981) | 36.60 (4.763) | 35.21 (4.730) |
| *σ* = 5 | 40.51 (5.151) | 427.77 (79.085) | 33.09 (4.634) | 32.60 (4.761) |
